# Impact of Radiation Quality on Microdosimetry and Chromosome Aberrations for High-Energy (>250 MeV/n) Ions

**DOI:** 10.3390/life12030358

**Published:** 2022-03-01

**Authors:** Floriane Poignant, Ianik Plante, Luis Crespo, Tony Slaba

**Affiliations:** 1 National Institute of Aerospace, Hampton, VA 23666, USA; 2 KBR, Houston, TX 77058, USA; ianik.plante-1@nasa.gov; 3 NASA Langley Research Center, Hampton, VA 23681, USA; luis.g.crespo@nasa.gov (L.C.); tony.c.slaba@nasa.gov (T.S.)

**Keywords:** ionizing radiation, heavy ions, track structure, microdosimetry, chromosome aberrations

## Abstract

Studying energy deposition by space radiation at the cellular scale provides insights on health risks to astronauts. Using the Monte Carlo track structure code RITRACKS, and the chromosome aberrations code RITCARD, we performed a modeling study of single-ion energy deposition spectra and chromosome aberrations for high-energy (>250 MeV/n) ion beams with linear energy transfer (LET) varying from 0.22 to 149.2 keV/µm. The calculations were performed using cells irradiated directly by mono-energetic ion beams, and by poly-energetic beams after particle transport in a digital mouse model, representing the radiation exposure of a cell in a tissue. To discriminate events from ion tracks directly traversing the nucleus, to events from δ-electrons emitted by distant ion tracks, we categorized ion contributions to microdosimetry or chromosome aberrations into direct and indirect contributions, respectively. The ions were either ions of the mono-energetic beam or secondary ions created in the digital mouse due to interaction of the beam with tissues. For microdosimetry, the indirect contribution is largely independent of the beam LET and minimally impacted by the beam interactions in mice. In contrast, the direct contribution is strongly dependent on the beam LET and shows increased probabilities of having low and high-energy deposition events when considering beam transport. Regarding chromosome aberrations, the indirect contribution induces a small number of simple exchanges, and a negligible number of complex exchanges. The direct contribution is responsible for most simple and complex exchanges. The complex exchanges are significantly increased for some low-LET ion beams when considering beam transport.

## 1. Introduction

Galactic cosmic rays are composed of approximately 87% protons, 12% helium nuclei, and 1% high-charge and energy (HZE) ions [1]. They are ubiquitous in deep space, and difficult to shield, thus constituting one of the main limitations for the safety of missions beyond Low Earth Orbit. Long-term consequences of space radiation exposure include increased risk of radiation-induced cancer, cardiovascular disease, and cognitive impairment [2]. At the cellular scale, HZE ions have a pattern of energy deposition that is related to ion linear energy transfer (LET). This pattern is responsible for the induction of complex DNA damages that can lead to chromosomal aberrations, which are positively correlated with carcinogenesis [3,4]. Biological endpoints such as chromosome aberrations are instigated by direct energy depositions from tracks that intersect the cell nucleus and energy depositions from δ-electrons ejected from tracks that crossed neighboring cells. Such mechanisms are relevant to space radiation exposures from galactic cosmic rays and help in determining relative biological effectiveness factors extrapolated from dose–response curves at low dose. The ability to separate such energy contributions to different endpoints provides clear means of studying the track interactions.

Microdosimetry consists of studying energy deposition events in irradiated targets the size of a cell. Microdosimetry can be used to correlate inhomogeneous energy deposition events at the micro-scale (ion track structure), with biological outcomes such as chromosome aberrations [5], thus providing means to better understand the basic mechanisms of biological response to HZE ions. In a previous work [6], we used the Monte Carlo (MC) track structure code RITRACKS [7] to investigate the microdosimetric contribution of tracks directly crossing targets (direct contribution) compared to δ-electrons coming from neighboring tracks that did not intersect the target (indirect contribution) for mono-energetic ion beams of LET varying from 0.22 to 150 keV/μm. We considered high-energy ions (>250 MeV/n) that had a similar energy distribution of emitted electron, spanning from eV to 10^5^ eV. We calculated *f*(*ε*,*D*), defined as single-track spectra of energy deposition ε (eV) imparted to a spherical target exposed to a fixed irradiation dose *D* (Gy). It was obtained by summing all energy deposition events imparted to the target for each individual track. Our work showed that the indirect contribution accounted for up to 18 to 22% of the energy deposited, on average, per ion track regardless of beam characteristics. The direct contribution, in contrast, displayed a strong dependence to the ion LET and made up most of the track energy deposition. The indirect contribution displayed high probabilities of having low *ε* (<10^4^ eV), while the direct contribution induced significant probabilities of having larger *ε* (>10^4^ eV), and such probabilities increased with increasing LET.

This study aims at extending our previous work with two main objectives. First, we went one step further toward a more realistic ground-based experimental setup by considering the transport of the incident ion beam inside a digital model of a mouse (Digimouse) [8]. We did this by simulating the irradiation of the Digimouse with mono-energetic ion beams using the MC transport code Geant4 [9]. The particle transport led to realistic poly-energetic mixed ion spectra that represent the irradiation field experienced by a cell that is part of a mouse tissue. Given the large ion energies and the size of the animal, little variability is obtained across different tissues. Both mono-energetic and poly-energetic beams were used as an input for the Monte Carlo code RITRACKS to compare the outcome at the cellular scale. We then calculated direct and indirect track structure contributions to *f*(*ε*,*D*) for these poly-energetic spectra and studied the impact of beam transport and production of secondary particles on microdosimetry results as compared to mono-energetic ion beams alone. Second, we calculated the formation of both simple and complex chromosome aberrations using the MC code RITCARD [10], for both mono-energetic ion beams and the corresponding poly-energetic spectra, to correlate energy deposition patterns and radiation quality dependence to a measurable biological endpoint. As for microdosimetry, we also separated the direct and indirect contributions to the formation of chromosome aberrations.

## 2. Materials and Methods

### 2.1. Macro-Scale Approach

We considered the 6 mono-energetic ions listed in Table 1. The ion energies considered in this work are relatively high (≥250 MeV/n) and consequently have energy distribution of emitted electrons that are similar, ranging from a few eV up to more than 10^5^ eV [6]. To simulate the transport of ion beams in rodents (they are extensively used in ground-based radiobiology experiments [1]), we irradiated a digital mouse (Digimouse) with each of the mono-energetic ion beams separately, using the MC transport code Geant4 [9]. In a typical experimental setup such as that described in [11], mice are not physically constrained. During the irradiation, several mice are contained within a plastic holding box and allowed to move, resulting in mice having different orientations with respect to the facility beam. For the irradiation simulation of the (immobile) Digimouse, we thus used an isotropic beam orientation to approximate the random movement and variability across the different mice. The simulated poly-energetic spectra were then obtained by averaging ion-simulated fluences over intra-abdominal organs (bladder, stomach, spleen, pancreas, liver, and kidneys) as a body averaged surrogate. In recent work, this approach was used to calculate microdosimetry spectra and assess quality factors associated with intestinal and colonic tumorigenesis in APC^(1638N/+)^ male mice [12]. At the ion energies considered in this study, and given the small size of the Digimouse, dose distributions and fluences observed in the different organs were very similar. We used the average as a representative value of the fluence. 

### 2.2. Micro-Scale Approach

**Figure 1:**
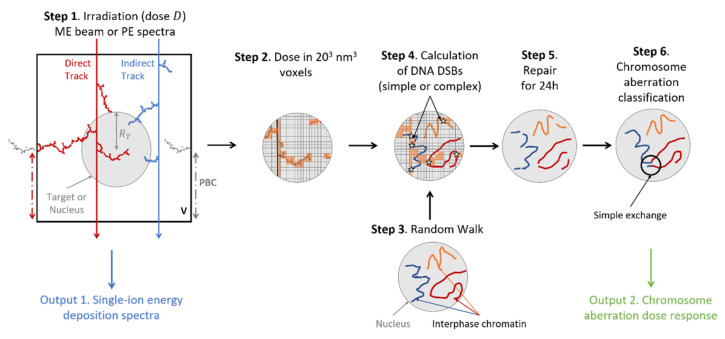
Scheme of the geometrical setup for the microdosimetry and chromosome aberration calculations.

Figure 1 presents the different steps that are performed to calculate single-ion energy deposition spectra and chromosome aberrations. The details are given in the Appendix A and only briefly overviewed here. In the first step, we simulated the irradiation to the spherical nucleus or target, of radius *R_T_* fixed to 4 μm and for a dose *D*, with either mono-energetic beams or poly-energetic spectra. To that end, we used the MC tool RITRACKS [7], which performs event-by-event tracking of energy deposition of ions in liquid water. As the δ-electrons ejected following interactions of ions with water molecules can have enough energy to travel several millimeters, we applied periodic boundary conditions (PBCs) to mimic the contribution of δ-electrons generated in neighboring volumes by tracks that have not intercepted the nucleus. Ion tracks were categorized as either direct when the ion path crossed the nucleus (red track on Figure 1), or indirect otherwise (blue track on Figure 1). The ions could either originate from the primary beam or be created by the interaction of the beam with the mouse tissues in case of poly-energetic beams. Single-ion energy deposition spectra normalized to the dose *D*, *f*(*ε*), were obtained at the end of this step, by summing all energy deposition events imparted to the target for each individual track. The total contribution, $f_{\mathrm{tot}}\left(\varepsilon \right)$, was broken down into direct ($f_{\mathrm{dir}}\left(\varepsilon \right)$) and indirect contributions ($f_{\mathrm{ind}}\left(\varepsilon \right)$) by summing energy deposition events due to direct or indirect tracks only. In this context, the direct and indirect contributions are different from the direct and indirect effects, which are terms commonly used in radiobiology for the damage to biomolecules by ionizations (direct effect) or resulting from the reactions of radical species (indirect effect).

Chromosome aberrations were computed with the RITCARD model [10,13,14] (Figure 1). Following the simulation of the nucleus irradiation (step 1), nanometric dose was scored in 20 × 20 × 20 nm^3^ voxels that mapped the cell nucleus (step 2). In parallel, a random walk algorithm was applied to model the 3D distribution of the 46 chromatin fibers contained in the nucleus during interphase (step 3). Next (step 4), the number of double-strand breaks (DSBs) was calculated by first locating intersection between interphase chromatin and voxels for which the energy deposited was greater than 0 eV. Then, the number of breaks in a voxel was sampled with a Poisson distribution, with parameter λ proportional to the energy deposited in that voxel. On average, the program yields ~35 DSBs/cell/Gy, with little dependence with ion LET [15,16]. The breaks were categorized as complex if the energy deposited in the voxel was greater than 500 eV, and simple otherwise. Next (step 5), a repair algorithm was applied over a time period of 24 h. Simple breaks followed an exponential decay with a short time constant (1.7 h) while complex breaks followed an exponential decay with a longer time constant (23.7 h) [17]. The algorithm proceeded in small time steps (typically 1 s), during which simple breaks could either be properly rejoined, or remained unrepaired. Additionally, complex breaks can be improperly repaired, leading to the formation of chromosome aberrations. The probability for mis-repair depended on the Euclidian distance between two complex breaks. Lastly, (step 6), chromosome aberrations were classified. In this work, we focused on simple and complex exchanges. Simple exchanges were defined as exchanges that involved 2 breaks in 2 chromosomes (dicentrics and translocations). This is illustrated on Figure 1. Complex exchanges were defined as exchanges that involved more than 2 breaks, in 2 or more chromosomes.

For a given ion beam, we calculated chromosome aberrations for 7 dose points ranging from 0.05 to 1 Gy. For each dose point, we simulated 10,000 MC histories. At the end of the simulation, for each dose point, we obtained an average number of exchanges and the standard error. As for microdosimetry calculation, we assessed the effect of direct and indirect contributions on chromosome aberrations. The estimation of the direct contribution to chromosome aberration yields was performed by scoring energy deposition in voxels due to direct tracks only, that is without scoring energy deposition due to indirect tracks. Likewise, the indirect contribution was performed by scoring energy deposition in voxels due to indirect tracks only. Thus, for a given beam, the simulation was performed 3 times to obtain the total, direct and indirect contributions. The dose response of simple or complex exchange frequency was then fitted by a linear quadratic (LQ) model,

(1) $$y_{i}\left(D_{av}\right)=\alpha_{i}D_{av}+\beta_{i}D_{av}^{2},$$

where $y_{i}\left(D_{av}\right)$ is the average number of exchanges (simple or complex) for the dose, *D_av_*, and contribution *i* (total, direct or indirect), for either mono-energetic beams or associated poly-energetic spectra. Note that *D_av_* represents the average dose obtained by RITRACKS at the end of a simulation. While for mono-energetic beams, this corresponds closely to the input dose *D*, we obtained a systematic deviation from the input dose *D* for poly-energetic spectra, from 1 to 10% depending on the beam energy. This was due to the extreme energies of the particle spectra, mainly low-energy heavy ion target fragments generated within Digimouse, in which the cross sections and LET values require further investigation. Nonetheless, these minor deviations are not expected to appreciably alter the results or conclusions of this work.

The procedure used to calibrate Equation (1) for the chromosome aberration dose response is described in the Appendix A. At the end of the procedure, we obtained a joint distribution for *α_i_* and *β_i_* values, out of which the average values, $\mu_{\alpha_{i}}$ and $\mu_{\beta_{i}}$, and the standard deviations, $\sigma_{\alpha_{i}}$ and $\sigma_{\beta_{i}}$, were computed. We also calculated the 95% prediction interval (PI).

Next, we wanted to assess whether there was a significant difference between chromosome aberration yields for mono-energetic beams vs. poly-energetic spectra to investigate the effect of beam transport within tissues. Similarly, to investigate possible interaction of breaks induced by the direct and indirect contributions, the total vs. direct + indirect contribution dose responses were compared. Breaks resulting from the direct and the indirect contributions might interact together and create aberrations that would not be accounted for when summing the two contributions. While beyond the scope of this work, an analysis of the results within the incremental effect additivity (IEA) framework, as applied elsewhere for mixed ion beams [18,19,20], could indicate possible synergy between tracks that directly cross the nucleus and delta-electrons of distant tracks.

To that end, we defined three statistical criteria, $m_{i\to j}$, $m_{j\to i}$ and $m_{KS}$, to compare the dose responses, as detailed in the supplemental file. They represent measures of agreement between the dose responses of contributions *i* and *j*. The quantities $m_{i\to j}$ and $m_{j\to i}$ are the probabilities for the contribution *i* (respectively *j*) to fall into the 95% PI of the contribution *j* (respectively *i*), integrated within the dose range 0–1 Gy. Values close to 1 indicate that dose responses *i* and *j* are not significantly different. The quantity $m_{KS}$ is the Kolmogorov–Smirnov statistic integrated between 0 and 1 Gy. Values close to 0 indicate that the two dose responses are statistically similar.

## 3. Results and Discussion

### 3.1. Mono-Energetic Beam vs. Poly-Energetic Spectra

**Figure 2:**
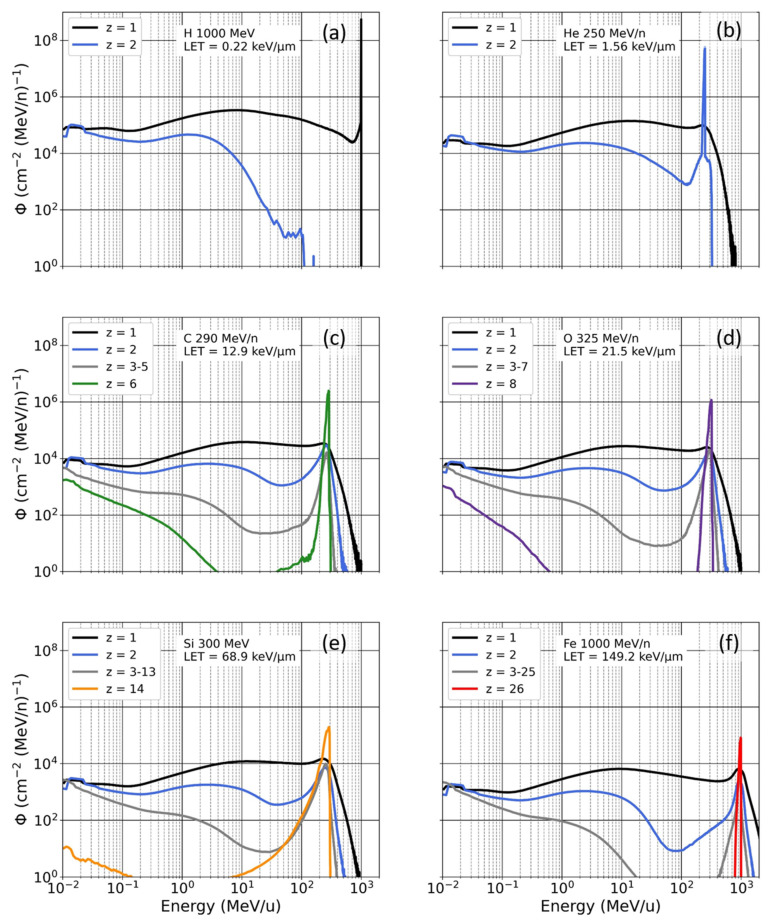
Poly-energetic beam fluence as a function of ion energy after the transport of mono-energetic beams ((**a**) H 1000 MeV, (**b**) He 250 MeV/n, (**c**) C 290 MeV/n, (**d**) O 325 MeV/n, (**e**) Si 300 MeV/n and (**f**) Fe 1000 MeV/n) in digital mice, averaged over intra-abdominal organs (bladder, stomach, spleen, pancreas, liver, and kidneys).

Figure 2 shows poly-energetic spectra of the fluence φ obtained by the Geant4 simulation as a function of ion energy. Each sub-figure shows the results obtained by irradiating the Digimouse with a mono-energetic beam, and the ion fluence is shown for atomic numbers varying from 1 to 26. The poly-energetic spectra are normalized so that the dose obtained by integrating the fluence over all energies and summing for all ions is 1 Gy. The calculated poly-energetic spectra show two features: A peak corresponding to the primary beam and a broad spectrum of secondary ions with majority of H and He ions, due to inelastic interactions between beam ions and tissue atoms. Overall, the beam fluence is dominated by the primary beam, with fluence peaking for the primary ion type and energy (e.g., H peaking at 1000 MeV for the 1000 MeV H mono-energetic beam) and reaching values approximately one (high-LET mono-energetic beam) to two (low-LET poly-energetic beam) orders of magnitude higher than those reached by secondary nuclei. For the Si beam, we observe a tail for the primary ions at lower energies due to the slowing down of the primary beam. For the C and O beams, spectral components observed at lower energies are due to secondary nuclei produced from target fragmentation. Secondary nuclei have a broad energy distribution, that also display a peak at the energy of the primary beam.

**Figure 3:**
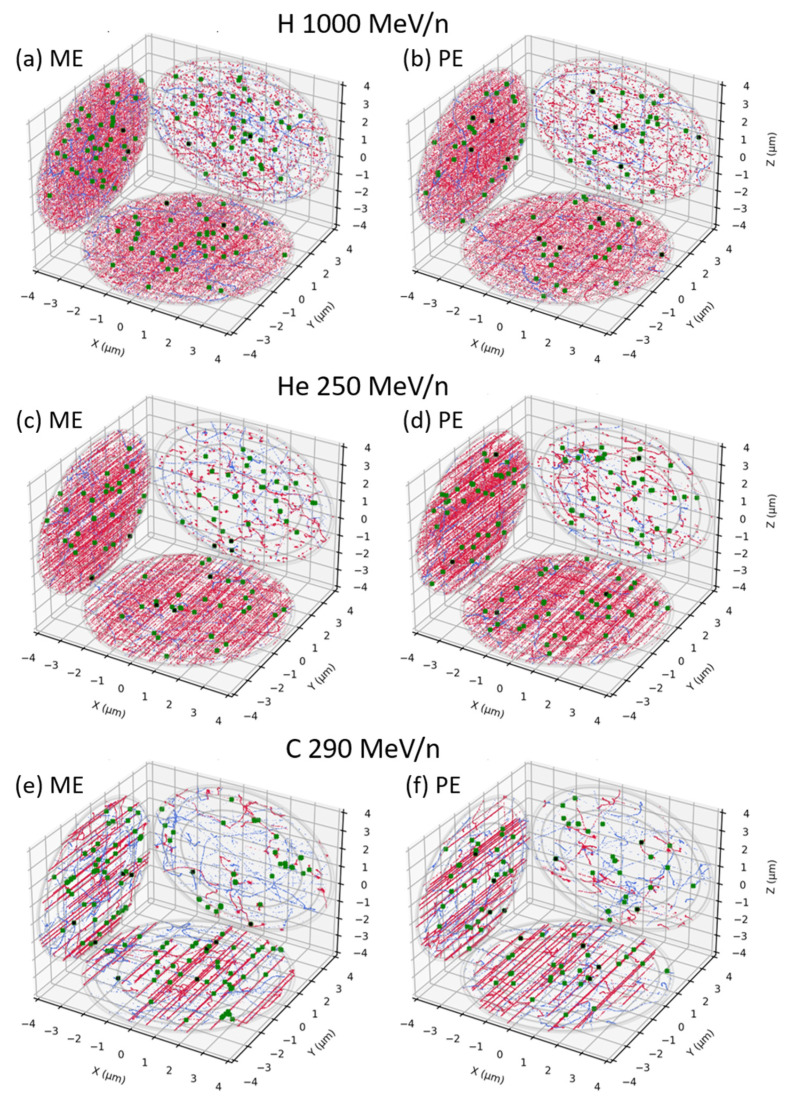
Examples of projected tracks and damages obtained for a dose of 1 Gy, for H 1000 MeV/n (**a**) + (**b**), He 250 MeV/n (**c**) + (**d**) and C 290 MeV/n (**e**) + (**f**). Tracks were clipped to display only energy deposition events inside the nucleus. The direct contribution is displayed in red and the indirect contribution in blue. Simple breaks are represented in green and complex breaks in black. For each beam, the results for mono-energetic (ME) beams are shown on the left ((**a**,**c**,**e**)) and for poly-energetic (PE) beams on the right (**b**,**d**,**f**)).

**Figure 4:**
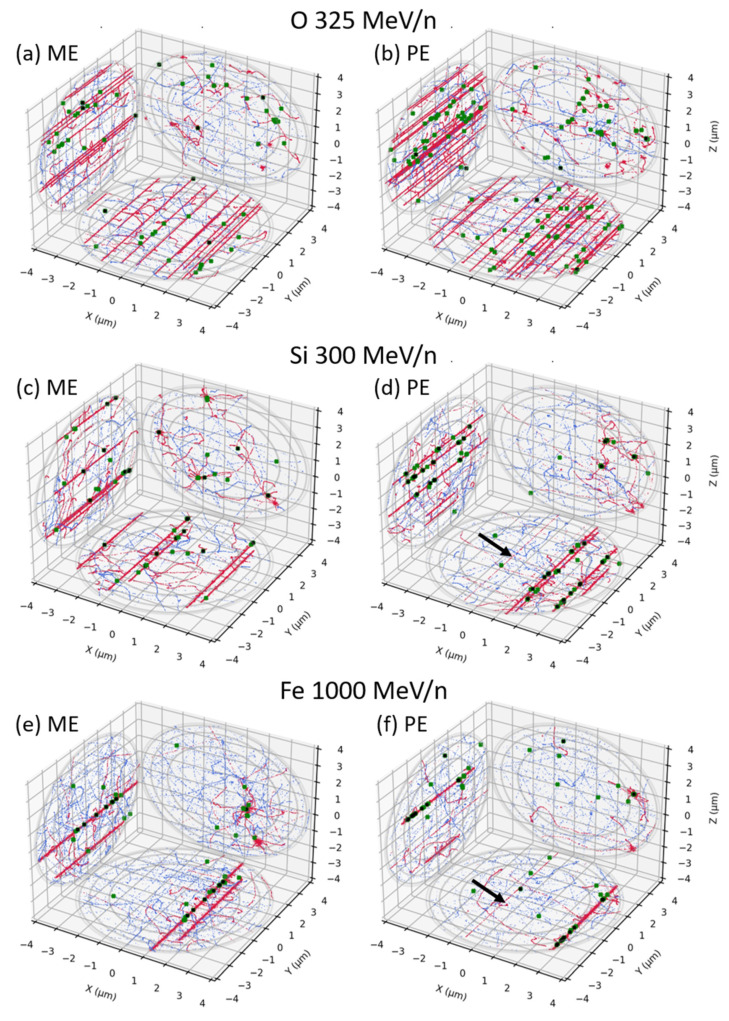
Same as Figure 3 but for O 325 MeV/n (**a**) + (**b**), Si 300 MeV/n (**c**) + (**d**) and Fe 1000 MeV/n (**e**) + (**f**).

Examples of tracks obtained within a cell nucleus by RITRACKS for the different beams are displayed in Figure 3 and Figure 4. Figures on the left are for mono-energetic beams directly impinging the cell nucleus, while figures on the right are for poly-energetic spectra, which correspond to the same mono-energetic beam but altered within the Digimouse. As we see in red, the energy deposition pattern of the direct contribution is highly dependent on the beam LET. The indirect contribution, displayed in blue, is due to δ-electrons and as such looks very similar from one beam to another. Note that the results for mono-energetic (left) and poly-energetic spectra (right) look very similar. However, some small differences are observable, in particular for high-LET beams, where we can see low-LET tracks crossing the volume for the direct contribution, as the black arrows point to in Figure 4. The figures also show examples of simple (green) and complex (black) break distributions. Both types of damages get clustered together along the tracks as the LET of the beam increases. The number of simple breaks is generally higher than that of complex breaks. However, the average number of complex breaks increases with increasing LET. The complex breaks are preferentially induced by the direct contribution, while the indirect contribution is responsible for simple, heterogeneously distributed, breaks. As for the tracks, the break distributions look similar for mono-energetic beams vs. the corresponding poly-energetic spectra inside Digimouse. However, on average, the number of complex breaks is slightly higher for the poly-energetic spectra, especially for those induced by low- to mid-LET beams. Note that these figures are only examples; the break distributions vary as one would expect from such stochastic simulations.

### 3.2. Microdosimetry

**Figure 5:**
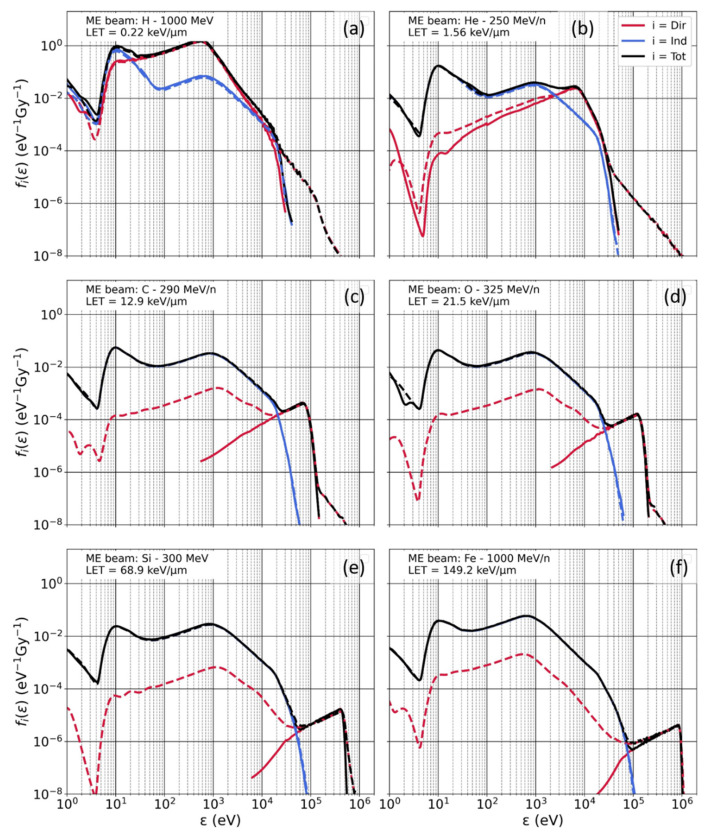
Single-ion energy deposition spectra, *f*_tot_(*ε*), in a spherical target. Results are displayed for 6 incident beams ((**a**) H 1000 MeV, (**b**) He 250 MeV/n, (**c**) C 290 MeV/n, (**d**) O 325 MeV/n, (**e**) Si 300 MeV/n and (**f**) Fe 1000 MeV/n), both with (poly-energetic spectra in dashed line) and without (mono-energetic (ME) beam in solid line) beam transport in the Digimouse. *f*_tot_(*ε*) (in black) is broken down into sub-contributions *f*_dir_(*ε*) (red) and *f*_ind_(*ε*) (blue).

Figure 5 shows single-ion energy deposition spectra, $f_{\mathrm{tot}}\left(\varepsilon \right)$, and sub-contributions,$\;f_{\mathrm{dir}}\left(\varepsilon \right)$ and $f_{\mathrm{ind}}\left(\varepsilon \right)$, for the six ion beams investigated in this study. The curves corresponding to mono-energetic beams (i.e., no beam transport in Digimouse) are shown as solid lines, whereas dashed lines correspond to poly-energetic spectra. Our previous work compared the results for mono-energetic beams with data from experimental and theoretical work [6].

As the figure shows, for mono-energetic beams, $f_{\mathrm{dir}}\left(\varepsilon \right)$ has a peak responsible for large single-ion energy deposition (*ε* > 10^4^ eV), except for very low-LET beams. Increasing the beam LET shifts the peak towards higher *ε* values. Conversely, $f_{\mathrm{ind}}\left(\varepsilon \right)$ shows little dependence on the beam LET, consistent with our previous work [6]. Indeed, as we previously showed, the ions considered in this work have high energies (≥250 MeV/n) and a similar energy distribution of emitted electrons. The indirect contribution is mostly due to longer-range (>few μm) δ-electrons that have thus similar energy deposition patterns regardless of the beam LET. The indirect contribution is responsible for low single-ion energy deposition (*ε* < 10^4^ eV) and represents ~18 to 22% of the single-ion energy deposition in the target, with the contribution increasing with increasing ion energy.

In general, results for poly-energetic spectra show that the indirect contribution is negligibly impacted by the transport of the beam in the Digimouse, when compared to results for mono-energetic beams. The energy distribution of δ-electrons shows minimal variation when accounting for beam transport. However, the direct contribution is affected by the transport of the beam within tissues in two ways when comparing it to results for mono-energetic beams. First, we observe an increase of $f_{\mathrm{dir}}\left(\varepsilon \right)$ for large *ε*, which is particularly significant for low-LET beams (1000 MeV H and 250 MeV/n He). This increase is mainly due to the production of low-energy heavy ions (i.e., target fragmentation) with high-LET during beam transport. While this is true regardless of the primary beam energy, such tendency is not as significant for high-LET beams since at high *ε*, the spectrum is dominated by the contribution of the primary beam. Second, we also observe that $f_{\mathrm{dir}}\left(\varepsilon \right)$ increases for lower *ε* values (i.e., *ε* < 10^4^ eV), resulting in $f_{\mathrm{dir}}\left(\varepsilon \right)$ having a broad distribution across *ε* rather than a peaked one. This is also explained by the production of secondary ions, but in this case, the secondaries are associated mainly with projectile fragments with mass and charge less than or equal to the primary beam. The energy and LET of these particles are broadly distributed as shown in Figure 2. Consequently, secondary low- to mid-LET ions can cross the target and lead to small amount of energy deposition that we do not observed for mono-energetic ion beams.

### 3.3. Chromosome Aberrations

**Figure 6:**
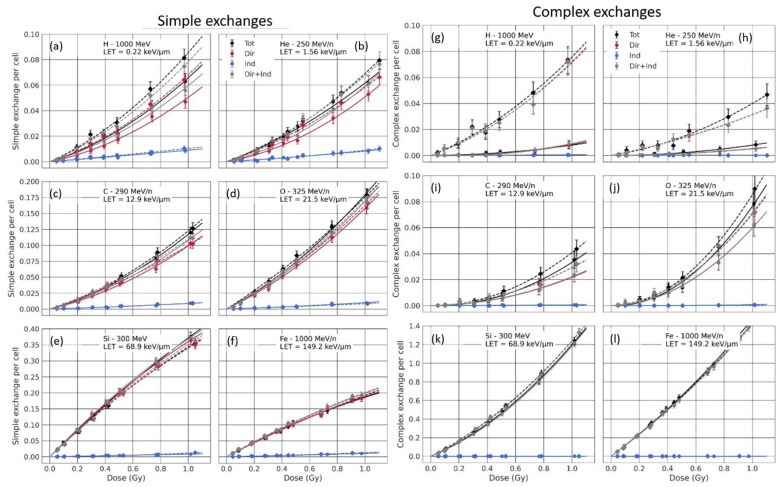
Simple (**a**–**f**) and complex (**g**–**l**) exchanges per cell for 6 incident beams (H 1000 MeV/n (**a**) + (**g**), He 250 MeV/n (**b**) + (**h**), C 290 MeV/n (**c**) + (**i**), O 325 MeV/n (**d**) + (**j**), Si 300 MeV/n (**e**) + (**k**) and Fe 1000 MeV/n (**f**) + (**l**)). Results are shown without beam transport (mono-energetic beam in solid line + round marker) and with beam transport in the Digimouse (poly-energetic beam in dashed line + diamond marker). The total (black), direct (red), indirect (blue) and direct+indirect (grey) were fitted with a linear quadratic model.

Figure 6 displays simple and complex exchanges per cell as a function of the dose, for different beams. Results in solid lines are for mono-energetic beams, while results in dashed lines are for poly-energetic spectra. The total number of exchanges (black) was broken down in direct (red) and indirect (blue) contributions. The sum of direct and indirect contributions (grey) was also displayed to compare it to the number of total exchanges and assess the effect of break interaction coming from the two contributions.

**Figure 7:**
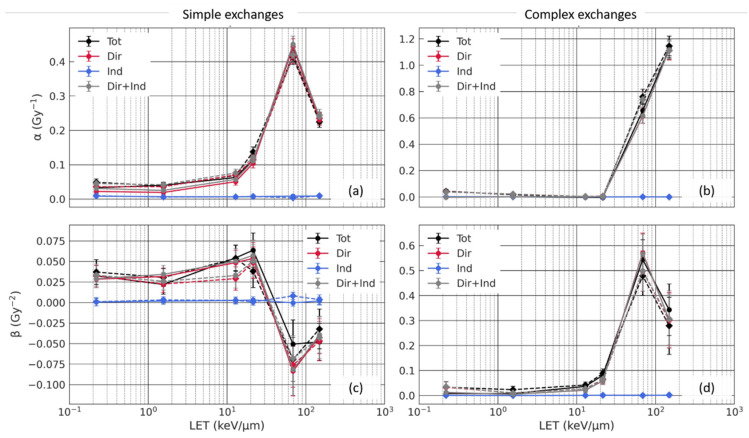
Average values of the LQ coefficients, *μ_α_* and *μ_β_*, for simple (**a**,**c**) and complex (**b**,**d**) exchanges as a function of the beam LET, both without beam transport (mono-energetic beam in solid line + round marker) and with beam transport in the Digimouse (poly-energetic spectra in dashed line + diamond marker). Error bars represent the standard deviation, *σ_α_* and *σ_β_*.

As Figure 6 shows, both simple and complex exchanges are well described by the LQ fit. For simple exchanges, we have positive β values for low to mid LET, followed by negative values at higher LET values (68.9 keV/µm and 149.2 keV/µm) due to a bending of the dose–response curve. Additionally, the number of simple exchanges at a given dose increases with increasing LET, except when the LET value goes from 68.9 keV/µm to 149.2 keV/µm. This can be explained by the fact that at high LET, breaks are created close together, increasing the probability of inter-chromosome exchanges, thus having a shift towards complex exchanges. As Figure 6 (right) shows, these high-LET values correspond to a sharp increase of complex exchanges. The trends are confirmed by Figure 7, which displays the average values of the α and β coefficients as a function of LET for both simple and complex damages, and the different sub-contributions.

Previous work showed that RITCARD could reproduce fibroblast simple exchange dose–response relationships for mono-energetic ion beams of LET spanning from 1.56 to 170 keV/µm [13] and for shielded ion beams [14]. Experimental dose responses for lymphocytes show similar trends [21]. The study reports simple and complex exchanges for Ti, Si, Ne, Fe and O ion beams of varying LET. For simple exchanges, α values peak for a LET ~40–120 keV/µm for simple exchanges with an apparent bending of the dose response in that LET range. For complex exchanges, α values usually increase with increasing LET, similarly to what we observe. An extensive benchmark considering available data for normal human cell lines [21,22,23,24] will be performed in future work.

#### 3.3.1. Analysis of the Sub-Contributions for Mono-Energetic Beams

When comparing the sub-contributions, we can see in Figure 6 and Figure 7 that while the indirect contribution induces a low but significant number of simple exchanges, it does not contribute significantly to complex exchanges. The direct contribution, on the other hand, induces most of simple and complex exchanges.

As shown in Table 2, the indirect contribution only induces 18 to 22% of the total energy deposition and is due to longer-range δ-electrons. Therefore, the number of indirect breaks is approximately 20% of the total number of breaks, while 80% is due to the direct contribution. This means that the probability of having more than one break (necessary for simple exchange) or more than two breaks (necessary for complex exchanges) in a nucleus is low for the indirect contribution. Additionally, the spatial distribution of indirect breaks is expected to be different than that of direct breaks, especially at high LET. As long-range δ-electrons that are responsible for the indirect contribution are low LET, the indirect breaks are sparsely distributed across the nucleus. This distribution does not change with beam LET as the energy distribution of δ-electrons in this work remains approximately the same, regardless of the beam energy, as all ions investigated have high energies comprised between 250 and 1000 MeV/n. Direct breaks, however, are more densely localized along ion tracks, and have a distribution that depends on the beam LET, as single-ion microdosimetry results showed. Such clustered distribution favors the formation of CA, since the misrepair probability increases with decreasing break distances. The number of indirect breaks is thus too low, and they are too sparsely distributed to induce a significant number of complex exchanges within this dose range and induce only a small number of simple breaks. Figure 7 shows that the corresponding *μ_α_* and *μ_β_* are independent of the beam LET. The direct contribution is thus responsible for the majority of the simple and complex exchanges, as Table 2 and Figure 6 and Figure 7 show. Moreover, the relative contribution of indirect simple exchanges at a fixed dose decreases for increasing LET. At 1 Gy, it is equal to 15.6% for a LET of 0.22 keV/μm, which is close to the relative indirect energy deposition. It slowly decreases as the LET increases and reaches only 5.2% for the highest LET (151 keV/μm).

**Figure A3:**
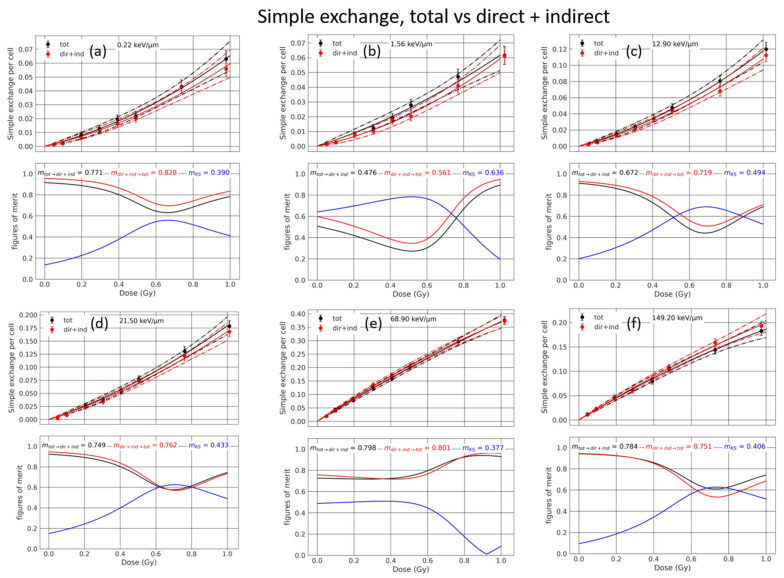
Dose–response comparison between the total vs. direct + indirect, for simple exchanges (upper figures). The figures of merit, $P_{\mathrm{tot}\to \mathrm{dir}+\mathrm{ind}}$, $P_{\mathrm{dir}+\mathrm{ind}\to \mathrm{tot}}$ and ${m^{\prime }}_{\mathrm{KM}}$, are plotted in black, red, and blue, respectively (lower figures). The values of the integrals over the dose (Equations (A18)–(A20)), $m_{\mathrm{tot}\to \mathrm{dir}+\mathrm{ind}}$, $m_{\mathrm{dir}+\mathrm{ind}\to \mathrm{tot}}$ and $m_{\mathrm{KM}}$ are indicated on each sub-figure. Results are displayed for (**a**) H 1000 MeV, (**b**) He 250 MeV/n, (**c**) C 290 MeV/n, (**d**) O 325 MeV/n, (**e**) Si 300 MeV/n and (**f**) Fe 1000 MeV/n.

**Figure A4:**
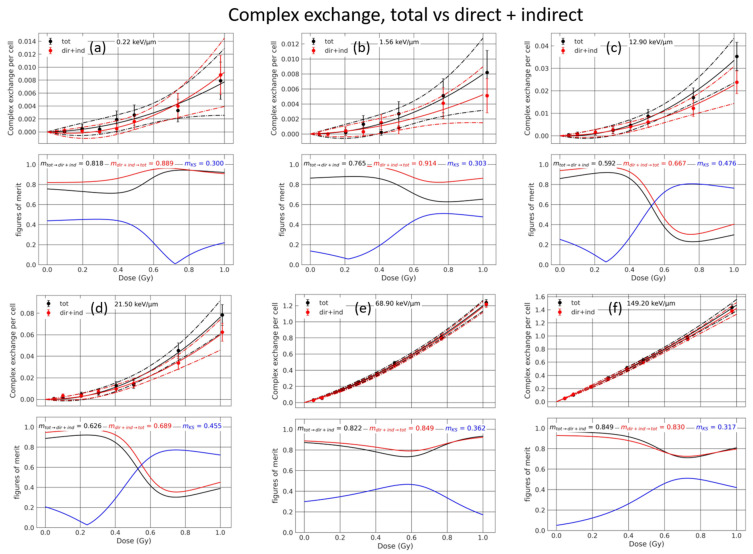
Dose–response comparison between the total vs. direct + indirect, for complex exchanges (upper figures). The figures of merit, $P_{\mathrm{tot}\to \mathrm{dir}+\mathrm{ind}}$, $P_{\mathrm{dir}+\mathrm{ind}\to \mathrm{tot}}$ and ${m^{\prime }}_{\mathrm{KM}}$, are plotted in black, red, and blue, respectively (lower figures). The values of the integrals over the dose (Equations (A18)–(A20)), $m_{\mathrm{tot}\to \mathrm{dir}+\mathrm{ind}}$, $m_{\mathrm{dir}+\mathrm{ind}\to \mathrm{tot}}$ and $m_{\mathrm{KM}}$ are indicated on each sub-figure. Results are displayed for (**a**) H 1000 MeV, (**b**) He 250 MeV/n, (**c**) C 290 MeV/n, (**d**) O 325 MeV/n, (**e**) Si 300 MeV/n and (**f**) Fe 1000 MeV/n.

When comparing chromosome aberration dose response for direct + indirect contribution as opposed to total contribution, we can see that they are overall very similar. Table 3 and Table 4 show the dose–response analysis, with *μ_α_* and *μ_β_* the mean values of the distribution of *α* and *β* parameters (Equation (1)) and *σ_α_* and *σ_β_* the standard deviations of that distribution. *μ_α_* and *μ_β_* are usually very close for total and direct + indirect contributions. However, slight differences could arise from the fact that breaks generated from indirect and direct energy depositions may interact together and form additional chromosome aberrations (either simple or complex) that we do not observe when simply adding the chromosome aberrations formed independently by the direct and indirect contributions. Indeed, we observe that for a few datapoints (e.g., complex exchanges for O 325 MeV/n and doses > 0.5 Gy as shown in Appendix A, Figure A3 and Figure A4), the number of chromosome aberrations for the total contribution appears to be greater than that for the direct + indirect contribution. In such cases, it is possible that breaks from the indirect contribution interact with breaks from the direct contribution and form complex exchanges that are not observed with the direct contribution alone.

One study hypothesized that DNA breaks of ion tracks directly crossing the nucleus, and those of δ-rays may interact together forming a curvature in the dose response of the total number of breakpoints involved in the formation of simple and complex exchanges [25]. δ-rays would thus add complexity to exchanges by involving a growing number of breakpoints. Our results, however, show a clear curvature for both simple and complex exchanges for the direct contribution, for all ranges of LET. This suggests that interaction of damages coming from two separate tracks could also play a role in the curvature of the dose–response relationship. To clarify this point, we plan in the future to extend RITCARD features to assess whether chromosome aberrations are formed due to breaks interacting from the same track, or from different tracks and, in particular, interaction of breaks coming from a track directly crossing the nucleus and breaks generated by δ-rays of neighbor tracks.

#### 3.3.2. Analysis of the Effect of Beam Transport

When comparing simple and complex exchanges for mono-energetic beams vs. poly-energetic spectra, we can see that the indirect contribution is not significantly affected by beam transport. This was expected as both microdosimetry and chromosome aberrations for different mono-energetic beams showed that this contribution did not depend on the LET, and thus the beam quality.

However, the number of simple and complex exchanges due to the direct contribution, and therefore the total contribution, are both significantly increased due to beam transport for some of the beams. While, as Figure 6 shows, this increase is relatively small for simple exchanges, it becomes quite significant for complex exchanges, especially for low-LET beams, as Figure 6 shows. Table 5 and Table 6 confirm such differences, with higher *μ_α_* and *μ_β_* values for poly-energetic spectra for complex exchanges a low-LET (≤1.56 keV/μm) values. This is consistent with microdosimetry single-ion energy deposition spectra, which showed a significant increase of high-energy deposition when accounting for beam transport. Such energy deposition patterns are well known for inducing efficiently complex exchanges. However, as Figure 7 shows, while this increase is significant for low-LET beams, the number of complex exchanges remains relatively small compared to the number of complex exchanges for high-LET beams (e.g., for Fe 1000 MeV/n).

## 4. Conclusions

This work investigated the effect of radiation quality and beam transport on both cell nucleus microdosimetry (single-ion energy deposition distribution) and chromosome aberrations by means of MC simulation with the radiation transport code RITRACKS/RITCARD. The effect of beam quality was assessed by considering mono-energetic ion beams of various LET and high energy (in the range 250 to 1000 MeV/n) and simulating their transport within a digital mouse to replicate experimental conditions of ground-based studies. The simulations yielded poly-energetic spectra obtained by averaging ion fluences over intra-abdominal organs as a reasonable body averaged surrogate. We distinguished two contributions: the direct contribution, due to energy deposition by ion tracks directly traversing the cell nucleus, and the indirect contribution, due to δ-electrons created by tracks traversing neighbor cells.

We show that for microdosimetry results and given the high-energy ion beams used in this study, the indirect contribution is not affected much by beam transport. The direct contribution, on the other hand, is highly dependent on the beam LET and thus is affected by beam transport. For low-LET ions, we observed that when beam transport and physical interactions in the mouse model are accounted for, there is a significant increase in the probability of having high-energy deposition events (>10^4^ eV) attributable to low-energy, high-LET nuclei produced by inelastic interactions between the incident beam and the mouse tissues. For high-LET ions, we found that the energy distribution changes from a peaked distribution (for mono-energetic beams) towards very high-energy deposition (>10^4^ eV), to a peaked distribution with a tail in the low-energy deposition range (<10^4^ eV). This tail is due to the production of low- to medium-LET secondaries produced in the mouse phantom from inelastic interactions.

For chromosome aberration predictions, we see that the number of simple exchanges due to the indirect contribution is low, and the number of complex exchanges is almost negligible. This is consistent the fact that the indirect contribution is responsible for about 20% of the energy deposition in the nucleus and is dominated by long-range, low-LET δ-electrons. On the contrary, the direct contribution is responsible for most of the simple and complex breaks. When considering beam transport within the digital mouse, we found a small but non-negligible increase in simple and complex exchanges that is particularly important for low-LET beams. While the final chromosome aberration yields induced by these low-LET poly-energetic beams remain much lower than those of higher-LET particles, this is important to consider it, since galactic cosmic rays are mostly composed in majority of proton and helium ions.

These model results provide important insight to help interpret experimental data and guide ongoing research efforts in the assessment of radiation quality. For ground-based radiobiology experiments involving rodents, observations are often attributed to the mono-energetic beam characteristics. This largely ignores the impact of physical interactions that could occur in a mouse and influence biological outcomes. In the case of energetic light ion beams such as H and He, mouse tissue barely modifies the primary ion energies. Nuclear collisions can occur with moderate probability though and yield secondary heavy ions (tissue target fragments) with high LET. We show that these secondary ions have a pronounced impact on microdosimetry quantities and chromosome aberrations. For energetic heavy ion beams, mouse tissue can notably change the primary ion energies in some cases, and a spectrum of secondary ions can be produced from Z = 1 up to the charge of the primary beam. The impact of these physical interactions can be seen in microdosimetry quantities but may not influence biological outcomes significantly since the primary ions dominate energy deposition at the cellular scale.

In this study, we considered ions with relatively high energy. This new capability of RITCARD to discriminate indirect and direct contributions to chromosome aberrations and related endpoints can help investigate track structure effects, by considering beams of similar LET values but with different ion charges, for which the energy spectra of δ-electrons are very distinct. For instance, it could help interpret results published by Loucas and colleagues [25] on the implication of long-range δ-electrons in the positive curvature of the dose response of breakpoints making up exchange events.

## Figures and Tables

**Table 1:** List of mono-energetic ion beam properties investigated in this work. LET values were calculated elsewhere [6] and estimated ranges were obtained with SRIM (http://www.srim.org/. Accessed 19 August 2021).

Ion	H^+^	He^2+^	C^6+^	O^8+^	Si^14+^	Fe^26+^
Energy (MeV/n)	1000	250	290	325	300	1000
LET (keV/µm)	0.22	1.56	12.9	21.5	68.9	149.2
Range in water (cm)	322	37.6	16.4	14.6	7.3	27.4

**Table 2:** Relative contribution of the direct and indirect contributions to the dose, and simple and complex exchanges at 1 Gy. For exchanges, the contributions were compared to the sum of the two contributions rather than the total contribution as both may vary, to reach a ratio of 100%. Standard errors are displayed in parenthesis. *R* is the ion range in water.

					Simple		Complex	
*LET*	R (cm)	*H*	*D*_dir_ (%)	*D*_ind_ (%)	y(1 Gy)_dir_ (%)	y(1 Gy)_ind_ (%)	y(1 Gy)_dir_ (%)	y(1 Gy)_ind_ (%)
0.22	322	1426	78.6	21.4	84.4 (12.4)	15.6 (4.0)	95.5 (47.0)	4.5 (6.5)
1.56	37.6	201	81.5	18.5	86.4 (12.3)	13.6 (3.6)	100.0 (63.1)	0.0
12.9	16.4	24	81.5	18.5	91.8 (9.5)	8.2 (2.0)	97.5 (29.6)	2.5 (2.9)
21.5	14.6	15	81.2	18.8	94.4 (8.0)	5.6 (1.3)	98.9 (18.9)	1.1 (1.3)
68.9	7.9	5	81.7	18.3	97.8 (5.2)	2.2 (0.5)	99.9 (4.8)	0.1 (0.1)
149.2	27.4	2	79.1	20.9	94.8 (6.9)	5.2 (1.2)	100.0 (4.8)	0.0 (0.1)

**Table 3:** Dose–response analysis for simple exchange (ME), total vs. direct + indirect, as presented in Section 2.2 and the Appendix A.

	Total					Direct + Indirect							
*LET*	*μ_α_*	*σ_α_*	*μ_β_*	*σ_β_*	*R* ^2^	*μ_α_*	*σ_α_*	*μ_β_*	*σ_β_*	*R* ^2^	$m_{t\to d+i}$	$m_{d+i\to t}$	$m_{\mathrm{KS}}$
0.22	0.033	0.008	0.032	0.012	0.98	0.031	0.008	0.029	0.012	0.97	0.76	0.82	0.39
1.56	0.040	0.008	0.022	0.012	0.97	0.026	0.007	0.033	0.011	0.98	0.50	0.55	0.63
12.9	0.062	0.011	0.056	0.016	0.99	0.057	0.011	0.051	0.016	0.99	0.69	0.71	0.48
21.5	0.115	0.014	0.064	0.020	0.99	0.110	0.014	0.059	0.019	0.99	0.74	0.78	0.43
68.9	0.423	0.023	−0.052	0.032	1.00	0.452	0.022	−0.084	0.029	1	0.80	0.81	0.37
149.2	0.234	0.016	−0.048	0.022	0.99	0.239	0.016	−0.039	0.022	0.99	0.76	0.75	0.42

**Table 4:** Same as Table 3, but for complex exchange (ME), total vs. direct + indirect.

	Total					Direct + Indirect							
*LET*	*μ_α_*	*σ_α_*	*μ_β_*	*σ_β_*	*R* ^2^	*μ_α_*	*σ_α_*	*μ_β_*	*σ_β_*	*R* ^2^	$m_{t\to d+i}$	$m_{d+i\to t}$	$m_{\mathrm{KS}}$
0.22	0.001	0.004	0.007	0.006	0.82	−0.003	0.003	0.012	0.005	0.9	0.80	0.89	0.31
1.56	0.000	0.003	0.008	0.005	0.84	0.001	0.003	0.004	0.004	0.76	0.76	0.92	0.30
12.9	−0.003	0.007	0.036	0.011	0.96	0.001	0.006	0.021	0.009	0.94	0.62	0.72	0.45
21.5	−0.004	0.011	0.081	0.018	0.98	0.002	0.010	0.059	0.015	0.97	0.63	0.70	0.45
68.9	0.659	0.055	0.539	0.078	1	0.616	0.055	0.569	0.078	1	0.84	0.84	0.34
149.2	1.128	0.073	0.345	0.107	1	1.116	0.071	0.307	0.102	1	0.82	0.84	0.34

**Table 5:** Same as Table 3, but total simple exchanges, mono-energetic (ME) beams vs. poly-energetic (PE) spectra.

	Mono-Energetic					Poly-Energetic							
*LET*	*μ_α_*	*σ_α_*	*μ_β_*	*σ_β_*	*R* ^2^	*μ_α_*	*σ_α_*	*μ_β_*	*σ_β_*	*R* ^2^	$m_{\mathrm{ME}\to \mathrm{PE}}$	$m_{\mathrm{PE}\to \mathrm{ME}}$	$m_{\mathrm{KS}}$
0.22	0.034	0.008	0.032	0.013	0.98	0.048	0.009	0.038	0.014	0.98	0.15	0.13	0.89
1.56	0.040	0.009	0.021	0.012	0.98	0.039	0.008	0.030	0.011	0.99	0.79	0.79	0.34
12.9	0.062	0.011	0.055	0.016	0.99	0.068	0.012	0.055	0.017	0.99	0.86	0.81	0.36
21.5	0.115	0.014	0.064	0.020	0.99	0.136	0.014	0.039	0.019	0.99	0.76	0.76	0.40
68.9	0.423	0.020	−0.051	0.028	1	0.414	0.021	−0.071	0.028	0.99	0.51	0.51	0.61
149.2	0.235	0.017	−0.049	0.023	0.99	0.223	0.017	−0.021	0.026	0.99	0.85	0.82	0.31

**Table 6:** Same as Table 3, but total complex exchanges, mono-energetic (ME) beams vs. poly-energetic (PE) spectra.

	Mono-Energetic					Poly-Energetic							
*LET*	*μ_α_*	*σ_α_*	*μ_β_*	*σ_β_*	*R* ^2^	*μ_α_*	*σ_α_*	*μ_β_*	*σ_β_*	*R* ^2^	$m_{\mathrm{ME}\to \mathrm{PE}}$	$m_{\mathrm{PE}\to \mathrm{ME}}$	$m_{\mathrm{KS}}$
0.22	0.000	0.004	0.008	0.006	0.83	0.042	0.014	0.034	0.021	0.95	0	0	1.00
1.56	0.000	0.003	0.008	0.005	0.83	0.017	0.010	0.023	0.013	0.93	0.6	0.01	0.98
12.9	−0.003	0.007	0.037	0.011	0.96	−0.001	0.008	0.042	0.012	0.97	0.75	0.67	0.47
21.5	−0.004	0.011	0.081	0.017	0.98	−0.002	0.012	0.088	0.018	0.98	0.85	0.79	0.35
68.9	0.653	0.056	0.546	0.079	1	0.764	0.060	0.477	0.084	1	0.34	0.31	0.77
149.2	1.127	0.070	0.344	0.103	1	1.147	0.076	0.236	0.120	1	0.75	0.72	0.39

**Figure A1:**
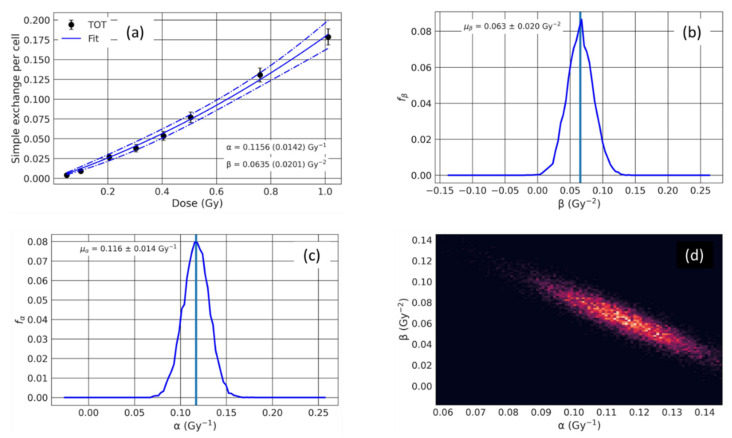
Linear quadratic fit for the dose response for mono-energetic O 325 MeV/n beam and simple exchanges. On the top left (**a**), dots are the results from RITCARD while the solid line represents the least squares fit, and the dashed line the 95% PI. The marginal PDF of the α and β are plotted on the top right (**b**) and bottom left (**c**), while the bottom right (**d**) shows samples of the joint density.

**Figure A2:**
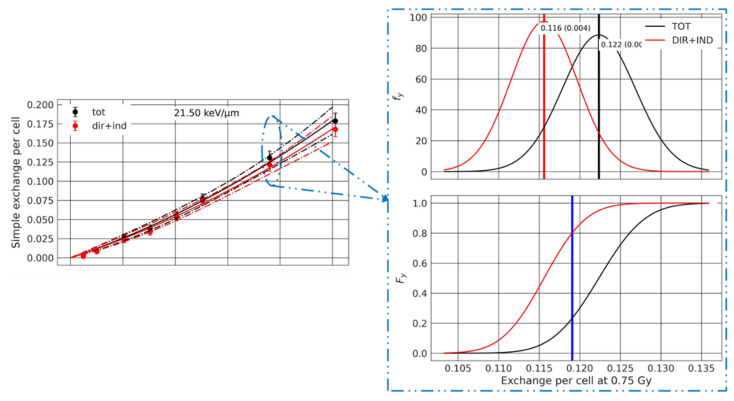
Illustration of the PDF, *f_y_* and CDF, *F_y_*, obtained at the dose point *D_av_* = 0.75 Gy, for the contributions *i* = tot and *j* = dir + ind, the mono-energetic beam O 325 MeV/n and simple exchanges. The vertical blue line represents the maximum of the difference between *F_y_*_,tot_ and *F_y_*_,dir+ind_.

**Figure A5:**
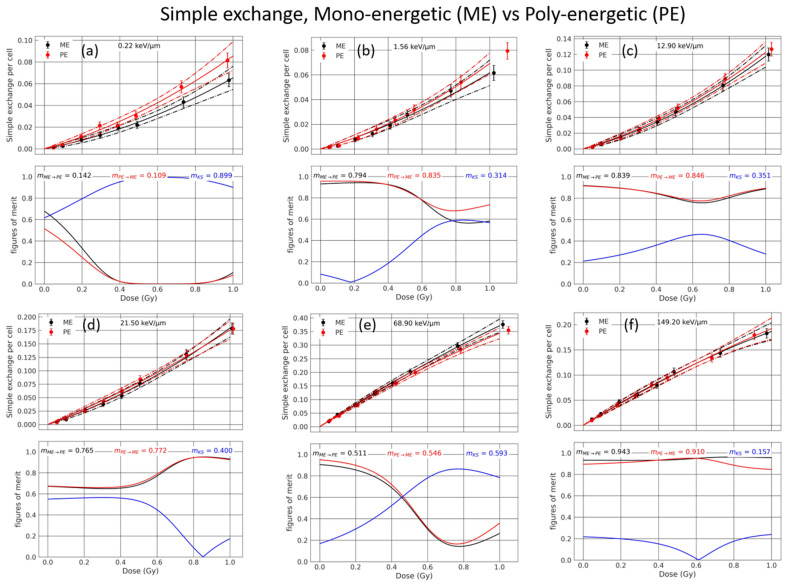
Dose–response comparison between the total contribution obtained for mono-energetic (ME) vs. poly-energetic (PE) beams, for simple exchanges (upper figures). The figures of merit, $P_{\mathrm{ME}\to \mathrm{PE}}$, $P_{\mathrm{PE}\to \mathrm{ME}}$ and ${m^{\prime }}_{\mathrm{KM}}$, are plotted in black, red, and blue, respectively (lower figures). The values of the integrals over the dose (Equations (A18)–(A20)), $m_{\mathrm{ME}\to \mathrm{PE}}$, $m_{\mathrm{PE}\to \mathrm{ME}}$ and $m_{\mathrm{KM}}$ are indicated on each sub-figure. Results are display for (**a**) H 1000 MeV, (**b**) He 250 MeV/n, (**c**) C 290 MeV/n, (**d**) O 325 MeV/n, (**e**) Si 300 MeV/n and (**f**) Fe 1000 MeV/n.

**Figure A6:**
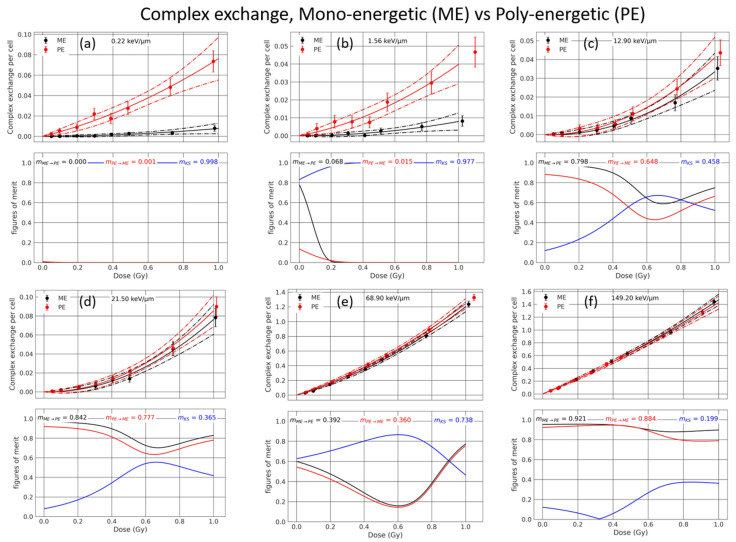
Dose–response comparison between the total contribution obtained for mono-energetic (ME) vs. poly-energetic (PE) beams, for complex exchanges (upper figures). The figures of merit, $P_{\mathrm{ME}\to \mathrm{PE}}$, $P_{\mathrm{PE}\to \mathrm{ME}}$ and ${m^{\prime }}_{\mathrm{KM}}$, are plotted in black, red, and blue, respectively (lower figures). The values of the integrals over the dose (Equations (A18)–(A20)), $m_{\mathrm{ME}\to \mathrm{PE}}$, $m_{\mathrm{PE}\to \mathrm{ME}}$ and $m_{\mathrm{KM}}$ are indicated on each sub-figure. Results are display for (**a**) H 1000 MeV, (**b**) He 250 MeV/n, (**c**) C 290 MeV/n, (**d**) O 325 MeV/n, (**e**) Si 300 MeV/n and (**f**) Fe 1000 MeV/n.

## Data Availability

The simulation results can be obtained by request to the corresponding author. The software RITRACKS that has been used to perform these calculations is available at https://software.nasa.gov (accessed on 19 January 2022).

## Appendix A. RITRACKS/RITCARD Simulation

### Appendix A.1. Simulation of Micrometric Volume Irradiation

RITRACKS [7] is a MC tool that simulates event-by-event energy deposition of ions of various energy and atomic numbers in liquid water, the main constituent of cells. It thus provides a detailed description of ion tracks at the sub-cellular scale and can thus be used for microdosimetry calculations or the study of DNA damages in the context of space radiation. In the present study, calculations were performed by defining a parallelepiped irradiation volume V encompassing a spherical volume of radius $R_{T}$, as depicted in Figure 1. The number of ions n crossing V is modeled as a Poisson distribution,

(A1) $$p\left(n\right)=\frac{\lambda^{n}\exp-\lambda }{n!}\;\;\;\;\;\;\mathrm{and}\;\;\;\;\;\;\;\;\lambda =\phi A,\;\;$$

where λ represents the average number of tracks traversing V, A is the surface of irradiation of V, and ϕ is the beam fluence obtained from the well-known equation,

(A2) $$D\;\left(\mathrm{Gy}\right)=1.6\times 10^{9}\phi \;\left({\mathrm{cm}}^{-2}\right)\;LET\;\left(\mathrm{keV}/\mu m\right),\;\;$$

where D is the irradiation dose, and the LET is obtained using Bethe’s equation with corrections [26]. For poly-energetic beams, the particle types and energies are obtained differently. The number of tracks of each ion type is calculated by numerically integrating the spectra,

(A3) $$\phi \left(Z\right)=\int_{E}\phi \;\left(Z,E\right)\;dE.\;\;$$

The contribution of each ion type to the dose is calculated as

(A4) $$D\left(Z\right)=1.6\times 10^{9}\int_{E}\phi \;\left(Z,E\right)\;LET\left(Z,E\right)dE.$$

The total dose is calculated by summing over each ion, i.e., $D_{tot}=\underset{Z}{\sum }D\left(Z\right)$. To simulate a given total dose, $D_{req}$, the fluences are multiplied by the ratio $D_{req}/D_{tot}$. The number of tracks for each Z is obtained by sampling the Poisson distribution using $\lambda =\left(D_{req}/D_{tot}\right)\phi \left(Z\right)A$. For each track, the energy is determined by using a rejection method. Essentially, a random energy $E_{rnd}$ is generated between $E_{min}$ and $E_{max}$, which are the minimum and maximum energies over which the spectra are defined. A random number U is drawn between 0 and the maximum value of the spectra for Z, $\phi_{max}\left(Z,E\right)$. If $U\leq \phi \left(Z,E_{rnd}\right)$, the energy value $E_{rnd}$ is accepted. The process is repeated until an energy is accepted.

For ions simulated in the present study, ejected δ-electrons have an energy distribution spanning between a few eV and hundreds of keV, with paths in tissue that can extend beyond a few millimeters [27]. Simulating such large volumes with RITRACKS would result in a prohibitive long calculation time. Thus, to model a realistic geometry of a cell located within a larger tissue structure, and therefore account for δ-electrons generated in neighboring volumes by tracks that may have missed the cell, we applied periodic boundary conditions (PBCs). PBCs are used to approximate large systems using a small, representative volume of space called the unit cell. As illustrated in Figure 1, when a secondary particle leaves the irradiated volume, it appears on the opposite side with the same velocity vector. Despite the use of PBC, the irradiation volume still must be set sufficiently large compared to the target volume to avoid simulation artifacts such as energetic δ-electrons crossing the boundary multiple times. Such artifacts would have negligible impact on single-track microdosimetric spectra but would influence total dose and resulting chromosome aberration yields. We analyzed the effect of irradiation volume size (not shown) and found that taking a side length for the irradiation area equal to 15 µm was large enough to avoid such artifacts.

### Appendix A.2. Single-Ion Energy Deposition Spectra

For the microdosimetry calculation, we irradiated V with a fixed dose D as presented in Appendix A.1. For each individual track, the sum of all energy deposition events imparted to the target, ε (eV), was calculated. This allowed us to compute single-ion energy deposition spectra f(ε;D). Dividing ε by the mean chord length allows to obtain the lineal energy. In this study, the radius of the target $R_{T}$ was fixed to 4 μm as a choice. Our previous work [6] showed that f(ε;D) scales with the target radius, and we thus expect to be able to extrapolate current results to other target sizes. Our calculations predict a complex dependence of chromosome aberration yields on the radius of the nucleus (not shown here) and does not scale directly with the nucleus. However, we expect that the main conclusions obtained in this work for the chromosome aberration direct or indirect contributions would still be valid for other nuclear geometries. The number of histories used for a given set of parameters varied from 10^3^ (low-LET beams) up to 10^6^ (high-LET beam). Raw histograms obtained with RITRACKS were analyzed using an adaptive Kernel Density Estimation approach [28]. As f(ε;D) scaled with the dose D, it was normalized to D so that

(A5) $$f\left(\varepsilon \right)=\frac{f\left(\varepsilon ;D\right)}{D}\;\;\mathrm{and},\;\;k\int_{0}^{\infty }\varepsilon \;f\left(\varepsilon \right)\;d\varepsilon =1\;\mathrm{Gy},$$

where f(ε) represents the energy deposition spectrum per single-ion track, normalized to the irradiation dose D and *k =* $1.6\times 10^{-19}\;\left(J\cdot {\mathrm{eV}}^{-1}\right)\times m_{T}\;\left(\mathrm{kg}\right)$ is a unit conversion factor, with $m_{T}$ the mass of the target.

To separate the direct and indirect contributions, an identification number was assigned to each track. When the axis of an ion track directly crossed the target, the energy deposition events associated with this track were counted as direct contribution (black track on Figure 1). Otherwise, it was counted as an indirect contribution (red track on Figure 1). The δ-electrons that were re-injected in the volume due to PBCs, as illustrated on Figure 1, were considered as indirect contribution, as they represent δ-electrons generated by neighboring tracks. These δ-electrons are also considered to be originating from a different ion track. The total, direct and indirect contributions are referred to as $f_{\mathrm{tot}}\left(\varepsilon \right)$, $f_{\mathrm{dir}}\left(\varepsilon \right)$ and $f_{\mathrm{ind}}\left(\varepsilon \right)$, respectively.

### Appendix A.3. Chromosome Aberrations

Chromosome aberrations were computed with the RITCARD model [10,13,14], which is briefly described next. RITCARD consists of different parts that are illustrated on Figure 1: (step 2) energy scoring in nanovoxels; (step 3) a random walk (RW) algorithm that simulates the geometrical distribution of chromosomes during interphase; (step 4) a DNA damage algorithm that assesses the number of double-strand breaks (DSBs); (step 5) a break repair algorithm; and (step 6) a function to categorize and count chromosome aberrations.

First, RITCARD requires the spatial map of energy deposition in the nucleus, as simulated by RITRACKS and explained in Appendix A.1. Once the tracks have been simulated for an irradiation dose D, nanometric dose was scored in 20 × 20 × 20 nm^3^ voxels that mapped the cell nucleus. In this study, the cell nucleus was of spherical shape with a radius of 4 μm to match the size of the target considered for microdosimetry calculations.

The RW algorithm was used to model the 3D position of all 46 chromosomes within the nucleus during interphase, as in [29,30]. Each chromosome was roughly modeled by a random coil and simulated by a sequence of monomers of lattice period of 20 nm, corresponding to the size of the dosimetry voxels. Each monomer contained 2 kbp of DNA. The initial position of the chromosome was sampled within a spherical chromosome domain [31] and the chromosome contained sub-structures representing chromosome loops of 60 monomers each.

The 3D voxel dose map and chromosome RW were then used to compute DSBs, by first locating intersections between chromatin fibers and voxels for which the dose was higher than 0 Gy. The number of DSBs N contained in a monomer was determined by sampling the Poisson distribution in Equation (A1) with,

(A6) λ=Q·D(i,j,k),  

where D(i,j,k) is the dose in the voxel of spatial coordinates (i,j,k) in lattice unit, and $Q=1.14\times 10^{-5}$ Gy^−1^ is an adjustable parameter representative of the intensity of DSB formation. The number of breaks in a monomer was rarely greater than 1, except in high-dose voxels in the core of high-LET tracks. On average, RITCARD yielded ~35 DSB/Gy/cell with little dependence with ion LET [15], as reported elsewhere [16]. Each break in a chromatin fiber led to the formation of two chromatin free ends.

The next part consisted of modeling break repair during the first 24 h after irradiation. The repair kinetics model was recently significantly updated [13,14]. It assumes that the number of breaks follows a bi-exponential decay as a function of time after irradiation,

(A7) $$N\left(t\right)=N_{1}\exp\left(-\frac{t}{\tau_{1}}\right)+N_{2}\exp\left(-\frac{t}{\tau_{2}}\right).\;\;$$

$N_{1}$, $N_{2}$, $\tau_{1}$ and $\tau_{2}$ are parameters. Such observations were reported by many investigators and suggests that simple breaks are repaired rapidly ($\tau_{1}$ = 1.7 h) while more complicated breaks take longer to repair ($\tau_{2}$ = 23.7 h) [17,32,33,34,35]. The time constants were set based on measured experimental constant times of fibroblasts [17]. $N_{1}$ and $N_{2}$ were not explicitly set, but breaks were categorized into simple and complex based on a voxel energy threshold of 500 eV. Using this threshold value, each free end associated with a given break was also categorized as either simple (voxel energy < 500 eV) or complex (voxel energy > 500 eV).

The repair algorithm proceeded by small time steps (typically 1 s) over a period of 24 h. At each time step, a repair attempt was made for all free ends. Each pair of simple free ends was assumed to repair properly (i.e., one free end recombined with the free end originating from the same DSB) or to remain unrepaired, with a probability of proper repair equal to $\delta t/\tau_{1}$. Complex free ends had an additional outcome, i.e., improper repair. For one complex free end, the total probability of proper and improper repair was $0.5\cdot \delta t/\tau_{2}$, with the 0.5 factor accounting for the fact that each complex free end was counted twice in the complex repair algorithm. If the free end was repaired during a time step, then the Euclidian distance, r, between the selected free end and all other complex free ends was calculated. The probability of any two ends repairing was then equal to,

(A8) $$I=\frac{1}{W}\exp\left(-\frac{r^{2}}{\sigma^{2}}\right).\;\;$$

W is an empirically calibrated parameter and $\sigma^{2}=0.8$ μm^2^ is an adjustable parameter. Equation (A8) reflects the fact that breaks further away from each other have a lower probability to recombine together. The algorithm then used the calculated probabilities to sample one free end for the selected break to repair with, thus leading to either proper or improper repair.

At the end of the 24 h period, the last part of RITCARD analyzed all the fragment sequences that were formed and classified them. The classification includes intact chromosomes, properly repaired chromosomes, and several types of chromosomes aberrations (translocation, inversions, deletions, dicentrics, rings and simple or complex exchanges). The criteria were defined by Ponomarev and colleagues [29,30] and are based on the work of [36]. Aberration types are not necessarily exclusive as, for example, a ring can also be a dicentric. In this work, we focused on simple and complex exchanges. Simple exchanges were defined as exchanges that involved two breaks in two chromosomes (dicentrics and translocations). This is illustrated on Figure 1. Complex exchanges were defined as exchanges that involved more than two breaks, in two or more chromosomes.

As for the microdosimetry calculation, we assessed the effect of direct and indirect contributions on chromosome aberrations. As described in Appendix A.2, tracks had an identification number to separate them into direct or indirect contributions. The estimation of the direct contribution to chromosome aberration yields was then performed by scoring energy deposition in voxels due to direct tracks only, which is without scoring energy deposition due to indirect tracks. The chromosome aberrations were then estimated, following the same steps as described above. Likewise, the indirect contribution was performed by scoring energy deposition in voxels due to indirect tracks only. Thus, for a given beam, the simulation was performed three times to obtain the total, direct and indirect contributions.

For a given ion beam, we calculated chromosome aberrations for 7 dose points ranging from 0.05 to 1 Gy. While for microdosimetry calculations, the number of histories depended on the beam energy. For chromosome aberration calculations, each dose point consisted of 10,000 histories. At the end of the simulation, for each dose point, we obtained an average number of exchanges and the statistical standard error.

### Appendix A.4. Dose–Response Statistical Analysis

The dose response of simple or complex exchange frequency was then fitted by a linear quadratic (LQ) model,

(A9) $$y_{i}\left(D_{av}\right)=\alpha_{i}D_{av}+\beta_{i}D_{av}^{2},$$

where $y_{i}\left(D_{av}\right)$ is the number of exchanges (simple or complex) for the dose $D_{av}$ and contribution i (total, direct, or indirect), for either mono-energetic or poly-energetic beams. For modeling purposes, $y_{i}$, $\alpha_{i}$ and $\beta_{i}$ are assumed to be continuous random variables.

A framework to optimally calibrate Equation (A1) according to random data is available in [37,38]. In this article, however, we carry out a suboptimal approach. Each dose–response datapoint $y_{i}\left(D_{av}\right)$ was sampled assuming that exchanges have a normal distribution, with a standard deviation equal to the simple or complex exchange statistical standard error. Random $y_{i}$ were drawn from this normal distribution and values of $\alpha_{i}$ and $\beta_{i}$ that minimize the least squares error were computed. This process was performed 1000 times, thereby leading to the data cloud of $\alpha_{i}$ and $\beta_{i}$ pairs shown in the bottom right of Figure 1. These data cloud were then used to learn a bivariate correlated normal using the maximum likelihood approach. This distribution is given by

(A10) $$\left[\begin{array}{c} \alpha_{i}\\ \beta_{i} \end{array}\right]\sim \;N\left(\left[\begin{array}{c} \mu_{\alpha ,i}\\ \mu_{\beta ,i} \end{array}\right],\;\left[\begin{array}{cc} \sigma_{\alpha ,i}^{2} & \rho \;\sigma_{\alpha ,i}\;\sigma_{\beta ,i})\\ \rho \;\sigma_{\alpha ,i}\;\sigma_{\beta ,i} & \sigma_{\beta ,i}^{2} \end{array}\right]\right),$$

where the first argument is the expected value, the second argument is the covariance, and ρ is the coefficient of correlation. This distribution, along with the simple structure of Equation (A9), make $y_{i}$ a normal distribution having the following closed form,

(A11) $$y_{i}\left(D_{av}\right)\;\sim \;N\left(\mu_{y,i}\left(D_{av}\right),\;\sigma_{y,i}^{2}=E\left[y_{i}{\left(D_{av}\right)}^{2}\right]-E{\left[y_{i}\left(D_{av}\right)\right]}^{2}\right),$$

where the expected value of $y_{i}\left(D_{av}\right)$ is

(A12) $$E\left[y_{i}\left(D_{av}\right)\right]=\mu_{y_{i}\left(D_{av}\right)}=\mu_{\alpha ,i}D_{av}+\mu_{\beta ,i}D_{av}^{2},$$

and the variance $\sigma_{y,i}^{2}$ is given by

(A13) $$E\left[y_{i}{\left(D_{av}\right)}^{2}\right]=\left(\mu_{\alpha ,i}^{2}+\sigma_{\alpha ,i}^{2}\right)D_{av}^{2}-2\left(\mu_{\alpha ,i}+\mu_{\beta ,i}+\rho \;\sigma_{\alpha ,i}\;\sigma_{\beta ,i}\right)D_{av}^{3}+\left(\mu_{\beta ,i}^{2}+\sigma_{\beta ,i}^{2}\right)D_{av}^{4},$$

with

(A14) $$E\left[y_{i}{\left(D_{av}\right)}^{2}\right]=E\left[\alpha_{i}^{2}\right]D_{av}^{2}+2\;E\left[\alpha_{i}\beta_{i}\right]D_{av}^{3}+E\left[\beta_{i}^{2}\right]D_{av}^{4},$$

and

(A15) $$\left[\alpha_{i}\beta_{i}\right]=E\left[\alpha_{i}\right]E\left[\beta_{i}\right]+\rho \;\sigma_{\alpha ,i}\;\sigma_{\beta ,i}.$$

For any fixed dose point, the 95% PI is given by

(A16) $$I_{i}=\left[\mu_{y,i}\left(D_{av}\right)-\eta \sigma_{y,i}^{2},\;\mu_{y,i}\left(D_{av}\right)+\eta \sigma_{y,i}^{2}\right]=\left[I_{i}^{-},\;I_{i}^{+}\right],\;$$

where η=1.96. Figure A1 presents an example of the results of such a procedure. Note that the parameter dependencies between α and β, which lead to a sizable value for ρ, play a key role.

Next, we want to assess whether there is a significant difference between dose–response curves for two cases: first $y_{\mathrm{tot}}$ for mono-energetic beam and $y_{\mathrm{tot}}$ for poly-energetic (Digimouse) beam to investigate the effect of beam transport; and second $y_{\mathrm{tot}}$ and $y_{\mathrm{dir}+\mathrm{ind}}$ for mono-energetic beam to investigate potential synergy due to the interaction of breaks induced by direct and indirect contributions. We thereafter refer to contributions i and j as those we compare to each other. To perform the comparison, we defined three figures of merit.

For a given dose point $D_{av}$, the probability of $y_{i}\left(D_{av}\right)$ for a contribution j to be contained in the 95 % PI, $I_{i},$ of the contribution i is,

(A17) $$P_{j}\left(I_{i}\;\left(D_{av}\right)\right)=P_{j}\left(I_{i}^{-}\left(D_{av}\right)\leq y_{j}\left(D_{av}\right)\leq I_{i}^{+}\left(D_{av}\right)\right)=F_{j}\left(I_{i}^{+}\left(D_{av}\right)\right)-F_{j}\left(I_{i}^{-}\left(D_{av}\right)\right),\;$$

where $F_{j}$ is the cumulative distribution function of the normal random variable as defined in Equation (A4). Note that $0\leq P_{j}\leq 1$ and the greater $P_{j}$, the more similar both contributions are. Considering a dose range of 0–1 Gy, the first two measures of agreement between the contributions i and j are

(A18) $$m_{i\to j}=\int_{0}^{1}P_{i}\left(I_{j}\left(D_{av}\right)\right)\;dD_{av},\;$$

(A19) $$m_{j\to i}=\int_{0}^{1}P_{j}\left(I_{i}\;\left(D_{av}\right)\right)dD_{av}\;$$

where the variables in Equation (A19) are analogous to those in Equation (A18). The closer the responses to both contributions, the closer $m_{i\to j}$ and $m_{j\to i}$ are to 1.

The third figure of merit is based on the Kolmogorov–Smirnov statistic. At a given dose point $D_{av}$, it is defined as the largest absolute difference between the two CDF of $y_{i}\left(D_{av}\right)$ and $y_{j}\left(D_{av}\right)$. The figure of merit is defined as the integral of this quantity over the dose range,

(A20) $$m_{\mathrm{KS}}=\int_{0}^{1}\underset{y}{\max}\left|F_{i}\left(y_{i}\left(D_{av}\right)\right)-F_{j}\left(y_{j}\left(D_{av}\right)\right)\right|dD_{av}=\int_{0}^{1}{m^{\prime }}_{\mathrm{KS}}\left(D_{av}\right)\;dD_{av}\;.\;$$

This metric is illustrated in Figure A2 below, by the vertical blue line indicating where the highest difference between the two CDFs is reached. Values of $m_{\mathrm{KS}}$ close to 0 indicate that the two dose responses are similar. All three figures of merit are free to take values between 0 and 1.

Figure A3, Figure A4, Figure A5 and Figure A6 show Equation (A8) as a function of the dose and the value of the corresponding figures of merit. For most figures, we observe that $P_{j}\left(I_{i}\;\left(D_{av}\right)\right)$ and $P_{i}\left(I_{j}\;\left(D_{av}\right)\right)$ are usually close to 1 across the whole dose range, in agreement with the Kolmogorov–Smirnov statistic is close to 0. There are however few cases, such as in Figure A6, where the dose responses are clearly distinct, and consequently $m_{i\to j}$ and $m_{j\to i}$ are ~0. We also observe some cases where we start seeing differences between the two contributions for high doses (>0.5 Gy).
